# Contact experience with people living with dementia and dementia‐related stigma domains among old‐old adults: A cross‐sectional study

**DOI:** 10.1002/pcn5.70399

**Published:** 2026-09-08

**Authors:** Suguru Shimokihara, Kosuke Yama, Kazuki Yokoyama, Hikaru Ihira, Keitaro Makino, Atsushi Mizumoto, Hideyuki Tashiro, Ryo Miyajima, Kiyotaka Shimada, Nozomu Ikeda

**Affiliations:** ^1^ Graduate School of Biomedical Sciences, Health Sciences Nagasaki University Nagasaki Japan; ^2^ Department of Occupational Therapy, School of Health Sciences Sapporo Medical University Sapporo Japan; ^3^ Faculty of Medicine Kagoshima University Kagoshima Japan; ^4^ N Field Home‐Visit Nursing Station Dune Sapporo Sapporo Japan; ^5^ Department of Physical Therapy, School of Health Sciences Sapporo Medical University Sapporo Japan; ^6^ Department of Health and Physical Education, Graduate School of Education Hokkaido University Sapporo Japan; ^7^ Major of Physical Therapy, Department of Rehabilitation, Faculty of Healthcare and Science Hokkaido Bunkyo University Eniwa Japan; ^8^ Ebetsu City Hospital Ebetsu Japan; ^9^ Graduate School of Health Sciences Sapporo Medical University Sapporo Japan

Dementia‐related stigma is a psychosocial barrier to timely help‐seeking, diagnosis, and social inclusion for people living with dementia (PLWD).^1^ Dementia‐related stigma is not a single construct but includes several dimensions.^2^, ^3^ Previous research has suggested that experiences of interacting with and learning about PLWD may be associated with lower levels of dementia‐related stigma.^4^ However, little is known about whether contact experience with PLWD is differentially associated with specific stigma domains among old‐old adults. This population is of particular interest because the prevalence of dementia increases markedly with advancing age, especially from the age of 75 years and older, making dementia a condition that may be increasingly relevant to their own life stage.^5^ Therefore, we examined the association between contact experience with PLWD and dementia‐related stigma domains among community‐dwelling old‐old adults. Clarifying these associations may help inform the design and targeting of stigma‐reduction strategies for old‐old populations.

This cross‐sectional study was based on data from a community‐based health check‐up conducted by Sapporo Medical University. Among 78 participants who attended the December 2024 face‐to‐face assessment, 72 adults aged 75 years or older were included in the analysis. Dementia‐related stigma was assessed using the Japanese short version of the Public Dementia Stigma Assessment Scale (PDSA‐J12), which includes four domains: avoidance, fear of labeling, person‐centeredness, and fear of discrimination.^3^ Each item is rated on a 5‐point Likert‐type scale from 1 (*strongly disagree*) to 5 (*strongly agree*), and each three‐item domain score ranges from 3 to 15. Higher scores indicate stronger stigma, except for the person‐centeredness domain, which was reverse‐coded because higher scores in the original scoring direction indicate more favorable attitudes; thus, higher reverse‐coded scores represent reduced person‐centered attitudes. Contact experience with PLWD was defined as having a family member or friend with dementia or having work experience involving interaction with PLWD. Participants were categorized into no‐contact experience and contact experience groups.

Participant characteristics were compared between groups using the Mann–Whitney *U* test or Fisher's exact test, as appropriate. The distributions of continuous variables and the potential influence of outliers were assessed by visual inspection of histograms. No extreme outliers were identified, and no observations were excluded because of outliers. Linear regression models were then used to examine the association between contact experience and each stigma domain. Crude models included contact experience only. Adjusted models included age, sex, education years, Japan Science and Technology Agency Index of Competence (JST‐IC)^6^ score, and 15‐item Geriatric Depression Scale (GDS‐15)^7^ score. These covariates were selected based on previous evidence of associations with public stigma. Age,^8^ sex,^9^ and education years^10^ were included as basic sociodemographic factors associated with dementia‐related stigma. JST‐IC was included as an indicator of higher level competence, including health‐related knowledge^11^ and social engagement,^12^ and GDS‐15 was included as an indicator of depressive symptoms.^13^ The study was approved by the Ethics Committee of Sapporo Medical University (approval number: 28‐2‐7), and all participants provided written informed consent. All analyses were performed using R version 4.2.2. Statistical significance was defined as a two‐sided *p* < 0.05. Because this study was exploratory, no adjustment for multiple comparisons across the four PDSA‐J12 domains was applied.^14^

The results of this study are shown in Figure 1. Of the 72 participants, 47 had no contact experience, and 25 had contact experience. Regarding the contact experience categories, 23 participants reported having a family member or close friend with dementia, four reported occupational experience involving interaction with PLWD, and two reported both types of contact experience. Compared with participants without contact experience, those with contact experience were older and had lower avoidance stigma scores. No significant between‐group differences were observed in the other three dementia‐related stigma domains. In crude linear regression models, contact experience was significantly associated with lower avoidance stigma (estimate = −1.18, 95% confidence interval [CI]: −2.22 to −0.13). However, after adjustment for covariates, this association was attenuated and was no longer statistically significant (estimate = −0.71, 95% CI: −1.85 to 0.42). Contact experience was not significantly associated with the other stigma domains in either the crude or adjusted models.

**Figure 1:**
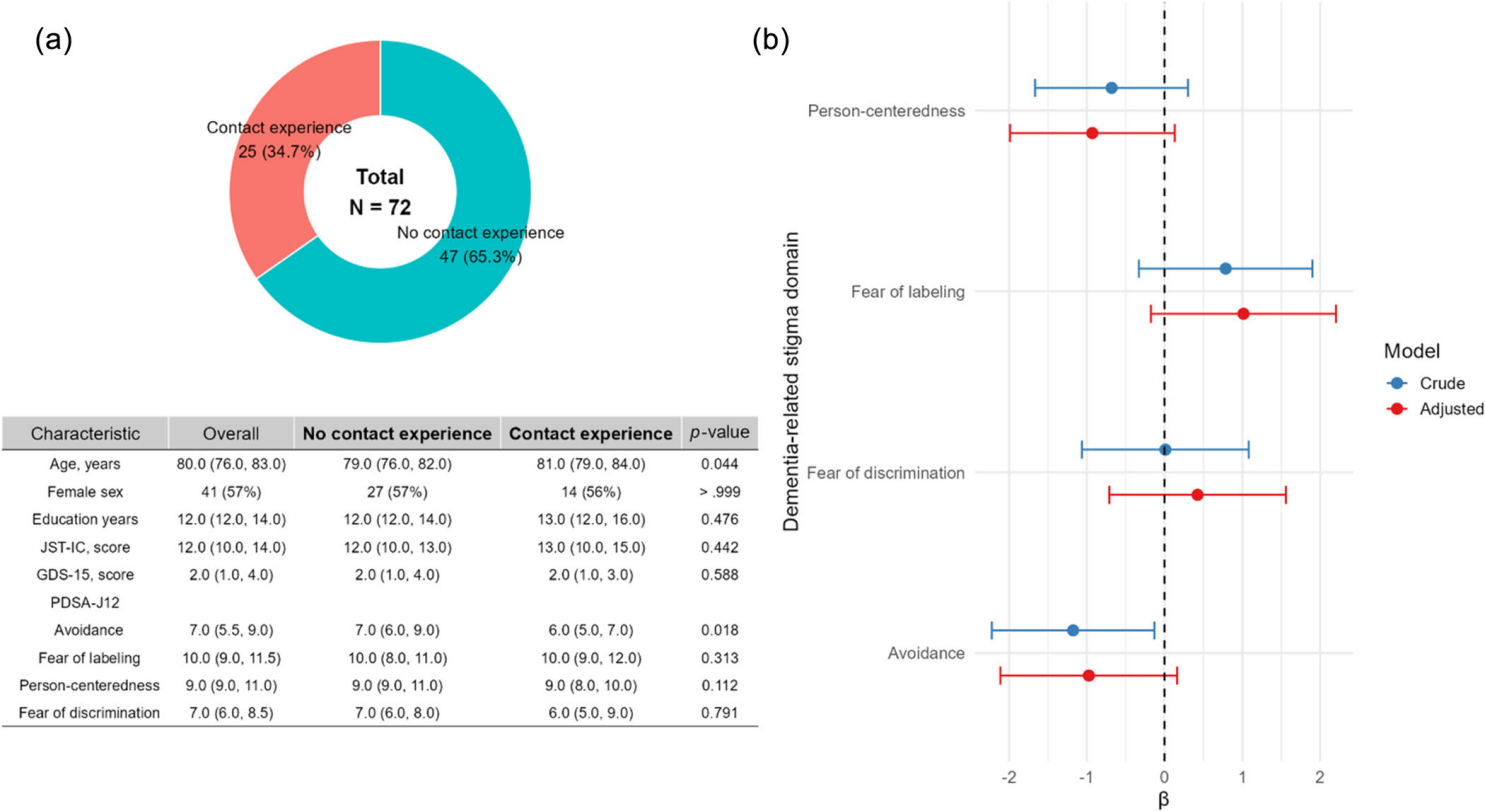
Associations between contact experience with people living with dementia and dementia‐related stigma domains. (a) Participant characteristics by contact experience of people living with dementia. Continuous variables are presented as medians with interquartile ranges, and categorical variables are presented as numbers and percentages. Between‐group comparisons were performed using the Mann–Whitney *U* test for continuous variables and Fisher's exact test for categorical variables. (b) Associations between contact with people living with dementia and stigma domains. Points represent unstandardized estimates, and horizontal bars represent 95% confidence intervals. Adjusted model: age, sex, education year, JST‐IC, and GDS‐15. β, unstandardized estimate; GDS‐15, 15‐item Geriatric Depression Scale; JST‐IC, Japan Science and Technology Agency Index of Competence; PDSA‐J12, Japanese short version of the Public Dementia Stigma Assessment Scale.

The crude findings suggest that old‐old adults who have contact experience with PLWD may show less avoidant attitudes toward dementia, while this association was no longer statistically significant after adjustment and should therefore be interpreted as preliminary. Contact experience may provide opportunities to perceive PLWD not as an abstract or threatening group but as individuals living in familiar social contexts. This interpretation is consistent with previous evidence suggesting that interaction experiences with PLWD are associated with lower levels of several dementia‐related stigma domains, particularly avoidance‐related attitudes.^4^ However, because the association was not statistically significant after adjustment, the relationship between contact experience and avoidance stigma among old‐old adults remains inconclusive. The attenuation after adjustment may reflect the influence of background characteristics, the small sample size, and the limited precision of the estimates.

This study has several limitations. The sample size was relatively small, and the cross‐sectional design precludes causal interpretation. Contact experience was assessed only by the presence or absence of family, friendship, or occupational contact experience, without evaluating the quality, frequency, closeness, or emotional valence of contact experience. We could not distinguish family contact experience from close‐friend contact experience, and contact experience type‐specific analyses were not conducted because the number of participants with occupational contact experience was very small. Moreover, participants were recruited from a health check‐up program in an urban setting and may have had relatively high health awareness. Finally, the adjusted models and domain‐specific findings should be interpreted as exploratory because some covariates may partly overlap with pathways linking contact experience and dementia stigma, and no adjustment for multiple comparisons was applied across the four PDSA‐J12 domains. Future studies should consider not only whether old‐old adults have contact experience with PLWD but also how such contact experience is experienced when designing and evaluating stigma‐reduction strategies.

## AUTHOR CONTRIBUTIONS

**Suguru Shimokihara** and **Kosuke Yama**: Conceptualization. **Suguru Shimokihara**: Formal analysis. **Suguru Shimokihara**: Funding acquisition. **Suguru Shimokihara, Kosuke Yama, Kazuki Yokoyama, Hikaru Ihira, Keitaro Makino, Atsushi Mizumoto, Hideyuki Tashiro, Ryo Miyajima** and **Kiyotaka Shimada**: Investigation. **Suguru Shimokihara, Kosuke Yama, Kazuki Yokoyama, Hikaru Ihira, Keitaro Makino, Atsushi Mizumoto, Hideyuki Tashiro, Ryo Miyajima** and **Kiyotaka Shimada**: Methodology. **Kazuki Yokoyama** and **Hikaru Ihira**: Project administration. **Kazuki Yokoyama** and **Hikaru Ihira**: Resources. **Nozomu Ikeda**: Supervision. **Suguru Shimokihara** and **Kosuke Yama**: Writing–Original draft. **Suguru Shimokihara, Kosuke Yama, Kazuki Yokoyama, Hikaru Ihira, Keitaro Makino, Atsushi Mizumoto, Hideyuki Tashiro, Ryo Miyajima, Kiyotaka Shimada** and **Nozomu Ikeda**: Writing—review and editing.

## CONFLICT OF INTEREST STATEMENT

The authors declare no conflict of interest.

## FUNDING INFORMATION

Japan Society for the Promotion of Science, Grant/Award Numbers: 24KJ1846 and 25K21134.

## ETHICS APPROVAL STATEMENT

This study was approved by the Ethics Committee of Sapporo Medical University (approval number: 28‐2‐7).

## PATIENT CONSENT STATEMENT

Written informed consent was secured from all participants prior to their inclusion in the study.

## CLINICAL TRIAL REGISTRATION

N/A.

## Data Availability

The data that support the findings of this study are available on request from the corresponding author, S.S. The data are not publicly available due to restrictions their containing information that could compromise the privacy of research participants.
